# Selective Chemical Activation of Piezo1 in Leukemia Cell Membrane: Single Channel Analysis

**DOI:** 10.3390/ijms22157839

**Published:** 2021-07-22

**Authors:** Valeria Vasileva, Elena Morachevskaya, Anastasia Sudarikova, Yuri Negulyaev, Vladislav Chubinskiy-Nadezhdin

**Affiliations:** Institute of Cytology, Russian Academy of Sciences, Tikhoretsky Ave. 4, 194064 St. Petersburg, Russia; vasileva.valeriia@gmail.com (V.V.); emorachevskaya@gmail.com (E.M.); anastasia.sudarikova@gmail.com (A.S.); yurineg@incras.ru (Y.N.)

**Keywords:** Piezo1, human leukemia cells, Yoda1, single channel patch-clamp

## Abstract

Piezo1/2 are mechanosensitive calcium-permeable channels that can be activated by various modes of membrane deformation. The identification of the small molecule Yoda1, a synthetic Piezo1 agonist, revealed the possibility of chemical activation of the channel. Stimulating effects of Yoda1 on Piezo1 have been mainly documented using over-expressing cellular systems or channel proteins incorporated in artificial lipid bilayers. However, the activating effect of Yoda1 on native Piezo1 channels in the plasma membrane of living cells remains generally undefined, despite the increasing number of studies in which the agonist is utilized as a functional tool to reveal the contribution of Piezo1 to cellular reactions. In the current study, we used the human myeloid leukemia K562 cell line as a suitable model to examine chemically induced Piezo1 activity with the use of the patch-clamp technique in various specific modes. The functional expression of Piezo1 in leukemia cells was evidenced using a combinative approach, including single channel patch-clamp measurements. Utilizing our established single-current whole-cell assay on K562 cells, we have shown, for the first time, the selective real-time chemical activation of endogenously expressed Piezo1. Extracellular application of 0.5–1 µM Yoda1 effectively stimulated single Piezo1 currents in the cell membrane.

## 1. Introduction

Cells and tissues are constantly exposed to various mechanical stimuli, and the fundamental process associated with the conversion of mechanical stresses to biological responses is called mechanotransduction. Mechanical forces could trigger different signaling pathways that regulate crucial cellular processes such as proliferation, differentiation, migration, gene expression and others [1]. The main actors in cellular mechanotransduction are mechanosensitive ion channels in plasma membranes due to their ability to provide rapid responses to mechanical forces, including the generation of a specific influx of calcium ions, which are known as ubiquitous second messengers in living cells [2]. The molecular identity of mechanosensitive calcium-permeable (MSCa) ion channels in mammalian cells remained obscure for a considerable period. Several molecular correlates were proposed as MSCa channels; however, the data were rather controversial [3,4]. In 2010, a breakthrough in the field of mechanotransduction was a discovery of a new family of MSCa channels: Piezo [5]. The Piezo family includes two members—Piezo1 and Piezo2—but despite a close homology, they have different biophysical properties as well as tissue distribution patterns and documented roles [6]. Piezo channels are widely expressed in cells and tissues, and their participation in a number of pathological processes, including oncotransformation, has been demonstrated [7,8].

Until recently, only various modes of membrane deformation were known as effective stimuli of mechanosensitive Piezo channels. In 2015, a heterocyclic compound Yoda1 (2-[(2,6-Dichlorophenyl) methylsulfanyl]-5-pyrazin-2-yl-1,3,4-thiadiazole) was reported to be the first selective chemical activator of Piezo1 (but, interestingly, not Piezo2, despite their close homology). Yoda1 has several related effects on Piezo1 gating, including the altering of channel kinetics, the tuning sensitivity of Piezo1 and the induction of channel activity in the absence of mechanical stimulation. Importantly, the activation of Piezo1 by Yoda1 does not require any cellular components other than a lipid bilayer [9]. In further experiments on various Piezo1 mutants overexpressed in HEK293 cells, it was proposed that Yoda1 activated the Piezo1 channel by directly binding to a specific protein domain located approximately 40 Å away from the channel pore [10]. Despite the suggested mechanism of Yoda1-induced Piezo1 activation, the stimulating effects of Yoda1 are mainly documented using over-expressing cellular systems or artificial lipid bilayers. Yoda1 is now widely used to reveal the functional impact of Piezo1 on various cellular processes and reactions. At the same time, it remains generally unknown how Yoda1 controls the activity of the endogenous Piezo1 channel in the plasma membrane. Moreover, the effective concentrations of Yoda1 needed to simulate ionic currents via Piezo1 in native cells remain undefined. Obviously, the unreasonably high concentrations of the agonist used in the studies could potentially lead to faulty conclusions on the degree of involvement of Piezo1 in cellular signaling.

In the present work, we utilized the human myeloid leukemia K562 cell line in which mechanosensitive regulations of ion channels were earlier investigated in electrophysiological experiments with the use of various patch-clamp configurations. Specifically, our previous patch-clamp investigations on K562 cells have documented the activity of native stretch-activated MSCa channels with biophysical properties close to the Piezo family [11,12,13]. Moreover, the leukemia K562 cell line presents an exclusive model to examine Yoda1-induced Piezo1 single currents in cell-attached patches as well as in the whole-cell membrane. The applied patch-clamp assay allowed us to monitor highly selective chemical Piezo1 activation in response to Yoda1. The detection of minimal effective concentrations of the agonist is critically important to search for the functional impact of Piezo1 activity on various physiological reactions in native cells. 

## 2. Results

**Figure 1:**
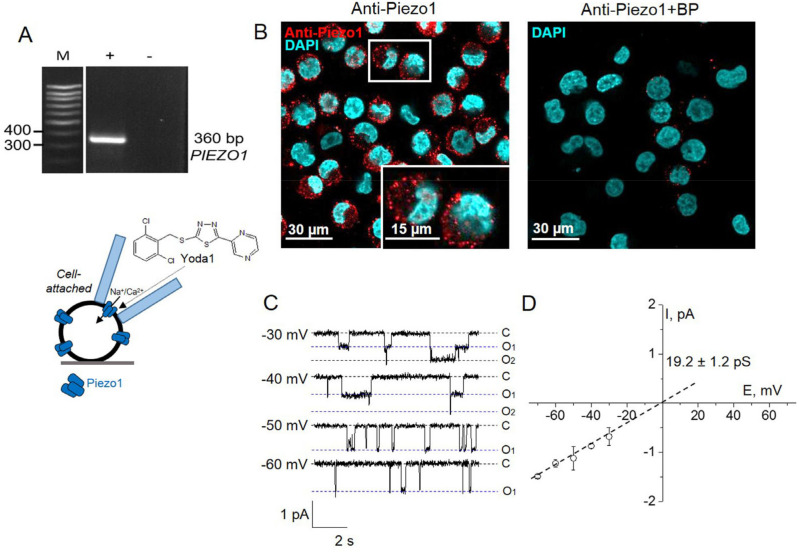
Piezo1 is expressed in human leukemia K562 cells. (**A**) RT-PCR analysis revealed the presence of *hPIEZO1* mRNA. Cropped gel with enhanced contrast is shown. (**B**) Immunofluorescent staining with specific antibodies detected Piezo1 proteins (Anti-Piezo1, red channel) in the cells. Cell nuclei were counterstained with DAPI (blue channel). Cells in white frame are shown in 2× zoom. No staining of the cells was observed after pre-incubation of the anti-Piezo antibody with the specific corresponding blocking peptide (Anti-Piezo+BP). (**C**) The single-channel activity of Piezo1 induced by Yoda1 (10 µM in the pipette solution) recorded in the representative cell-attached experiment at different membrane potentials. Here and elsewhere, the index shows a number of active channels (C—closed state (zero current), O—channel openings). Holding membrane potential is indicated near current traces. (**D**) The mean I-V relationship corresponds to a single-channel conductance of 19.2 ± 1.2 pS (*n* = 9).

Firstly, we used a specific transcriptome database (BioGPS, The Scripps Research Institute, La Jolla, CA, USA) to assess the level of *PIEZO1* gene expression in the human myeloid leukemia K562 cell line. Analysis of the resource indicated relatively high amounts of *PIEZO1* transcripts in K562 cells compared to the mean mRNA levels from an experimental dataset (119.3 vs. 12.5 as a median value for other cell lines; [14]). To verify the functional expression of Piezo1 in leukemia K562 cells, we applied a combinative approach by performing RT-PCR, immunofluorescence microscopy and patch-clamp measurements. Particularly, we detected the presence of *PIEZO1* mRNA in K562 cells with a product size predicted for selected primers (approximately 360 base pairs, Figure 1A). Consistently, immunofluorescent staining confirmed the expression of Piezo1 in K562 cells (Figure 1B). 

Further, various modes of patch-clamp technique were utilized to record and characterize Yoda1-induced single currents via native Piezo1 in the cell membrane. For the initial set of electrophysiological experiments, we used a canonical cell-attached configuration to analyze channel activation in response to Yoda1 application to the extracellular membrane surface. In control cell-attached patches (without agonist, *n* > 50), no significant spontaneous channel activity was observed. The inclusion of Yoda1 in the pipette solution allowed us to record the agonist-induced activity of Piezo1 channels. Figure 1C shows the representative recordings of single Piezo1 currents in the membrane fragment in the presence of 10 µM Yoda1 in the pipette. Corresponding unitary conductance was 19.2 ± 1.2 pS (*n* = 9); Erev was close to zero (Figure 1D). Chemically induced Piezo1 currents were found in 23 out of 47 stable cell-attached patches with Yoda1 in extracellular (pipette) solution. Our cell-attached experiments consistently evidenced the activating effect of Yoda1 on Piezo1 channels in K562 cells. To further validate Piezo1 activity in the cell line, another novel selective channel activator, Jedi2 [15], was utilized in our cell-attached experiments (Supplementary Figure S1). The inclusion of the Piezo1 activator Jedi2 in the patch pipette induced single-channel activity with the same biophysical characteristics as Yoda1-activated channels in K562 cells. The observed effects of two different selective agonists confirm the functional expression of Piezo1 in human leukemia K562 cells.

To explore the functional activity of Piezo1 in K562 cells, we designed specific whole-cell experiments in which single currents via Piezo1 were monitored in response to the real-time Yoda1 application. In these experiments, the pipette cytosol-like solution contained low [Ca^2+^]_i_ (pCa8, 10 nM, see Methods) that resulted in insignificant background channel activity; particularly, the absence of Ca^2+^-activated currents in the range of applied holding membrane potentials. These conditions allowed us to obtain almost ‘silent’ whole-cell patches on K562 cells in control before agonist application. Developed electrophysiological assay practically combines the known advantages of outside-out mode (recording of single currents) with the evaluation of channel activity in the whole-cell membrane. 

**Figure 2:**
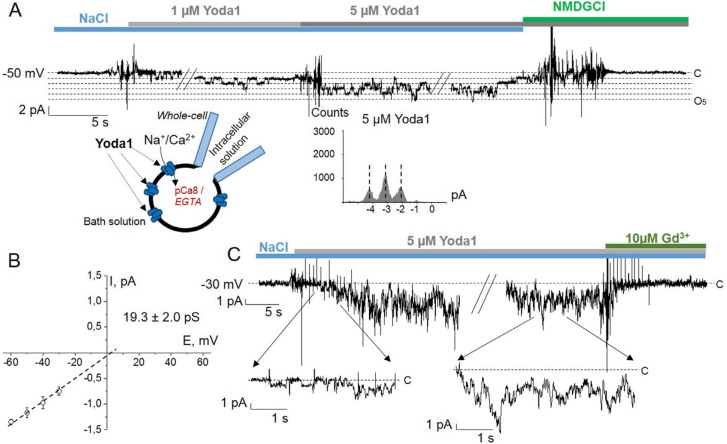
Single-channel recordings of Piezo1 activity in representative whole-cell experiments on K562 cells. (**A**) Extracellular application of Yoda1 resulted in the activation of Piezo1 in the whole-cell membrane. The replacement of all permeable cations with non-permeable NMDG^+^ resulted in the full abolition of Yoda1-induced ion currents. The amplitude histogram calculated for the particular current trace is presented below. Single-channel Piezo1 amplitudes could be clearly resolved between the centers of the peaks of the histograms. (**B**) The I-V relationship of Piezo1 channels. The mean unitary conductance of the channels is 19.3 ± 2.0 pS (*n* = 5). (**C**) The development of a rather high level of Piezo1 activity in the whole-cell membrane in response to 5 μM Yoda1 application. The parts of the traces indicated by arrows are shown in extended timescale. The following addition of MS channel blocker Gd^3+^ (10 µM GdCl_3_) to the bath resulted in the inhibition of Piezo1 activity. In (**A**) and (**C**), the substitutions of the extracellular solutions could clearly be seen.

For the initial set of whole-cell experiments, 5 µM of Yoda1 was chosen based on our cell-attached measurements of K562 cells (Figure 1C) as well as the published data on human Piezo-transfected HEK293T cells [9]. The addition of Yoda1 to standard Na^+^-containing extracellular bath solution induced Piezo1 activation in a time-dependent manner: maximal level of the activity was established in 10-15 s after the application of the agonist (Figure 2A,C). Whole-cell single-current recordings showed that the application of 5 µM Yoda1 to the bath solution induced the development of rather high Piezo1 activity in a part of experiments (Figure 2C) that may generally complicate the quantitative analysis of single-channel openings. Activation of Piezo1 by Yoda1 was observed in all whole-cell patches on K562 cells (*n* = 39); the integral Piezo1 activity in response to Yoda1 varied between the experiments. From a limited number of whole-cell experiments (with relatively low Piezo1 activity; see, for example, the representative full-trace currents and corresponding amplitude histograms in Figure 2A), single-channel conductance (Figure 2B) was estimated as 19.3 ± 2.0 pS (*n* = 5), which was identical to Yoda1-evoked cell-attached currents (Figure 1C). Importantly, the addition of gadolinium (10 µM GdCl_3_), a well-known inhibitor of MSCa channels, to the bath blocked Yoda1-induced Piezo1 activity (Figure 2C). Additionally, the replacement of all permeable cations with non-permeable NMDG^+^ resulted in the full abolition of ion currents (Figure 2A). Functional Piezo1 activity evoked by Yoda1 in leukemia cell membrane was additionally evidenced in whole-cell experiments with spider toxin GsMTx4, a known Piezo channel inhibitor ([16], Supplementary Figure S2). 

**Figure 3:**
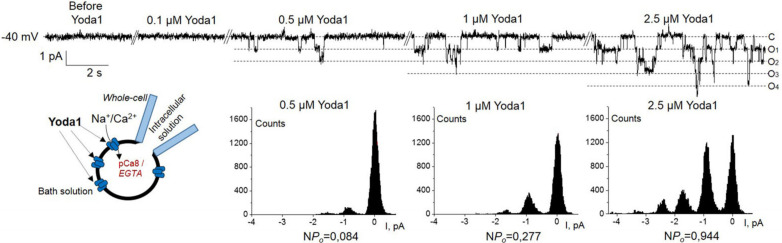
The detection of Piezo1 activity in response to submicromolar Yoda1 concentration. Representative whole-cell recording demonstrates single Piezo1 currents at various extracellular bath Yoda1 concentrations starting from 0.5 μM. Corresponding amplitude histograms and N*P_o_* values (calculated on presented intervals) are shown below the traces.

Further, whole-cell single-current recordings have been utilized to define the extracellular concentration of Yoda1 that would be sufficient to evoke single Piezo1 activity in the plasma membrane. We started with a very low (0.1 µM) concentration of Yoda1 [9] and progressively increased it over the course of our electrophysiological recordings. Figure 3 shows the development of the activity of Piezo1 channels in response to the gradual increase of Yoda1 concentrations applied to the cell in the representative whole-cell experiment (out of 5). Our observations indicate that distinct Yoda1-induced activity of Piezo1 could be detected already in response to 0.5 µM of the agonist. Further elevation of Yoda1 concentration progressively enhanced the activity of Piezo1 channels that is demonstrated by current records, corresponding amplitude histograms and N*P_o_* values (Figure 3). Whole-cell patch-clamp experiments have demonstrated highly selective chemical Piezo1 activation and allowed us to conclude that the concentration of Yoda 1 near 1 µM is sufficient to evoke stable single Piezo1 channel activity in the plasma membrane of K562 cells. Our single-channel whole-cell patch-clamp assay and relevant concentration range of extracellular agonists could be utilized in future studies to analyze Piezo1 permeation properties and potential blocking effects.

## 3. Discussion

We identified, for the first time, functionally active endogenous Piezo1 channels stimulated by the selective chemical agonist Yoda1 in human myeloid leukemia cells. Single-channel properties of Yoda1-induced currents were examined in cell-attached and whole-cell modes of the patch-clamp technique. The biophysical properties of chemically activated Piezo1 channels in leukemia K562 cells, particularly unitary conductance (near 19 pS) and Erev (0 mV) calculated from I-V relationships, were identical in both cell-attached and whole-cell experiments with Yoda1. The same values were obtained for stretch-induced currents in the parallel assay. These characteristics are rather similar to those reported previously for mammalian Piezo1 channels examined under comparable experimental conditions, particularly in native cells and heterologous overexpression systems. The most commonly observed values of unitary conductance were in the range of 20–30 pS [5,15,17,18,19]. Of note, there is an evident lack of single-current data in Piezo studies accompanied by obvious variability in reported conductance values. In a comprehensive electrophysiological study, Piezo1 conductance of about 15 pS was calculated from single-current measurements and the corresponding current-voltage relations in the wide range of membrane potential [20]. Conductance values of about 27–29 pS were reported for both Piezo1 and Piezo2 channels [19], implying that permeation properties can unlikely be used to identify the particular channel type. The selective activators such as Yoda1 and Jedi2 could be considered as more reliable tools to reveal the functional expression of Piezo1 channels in various cells. 

Importantly, MSCa channel activity with similar characteristics in response to plasma membrane stretch was documented in K562 cells [11,12,13,21], implying that MSCa channels in K562 cells could likely be at least partly identified as Piezo1 proteins. Earlier reports provided a comprehensive description of MSCa channels activated by the stretch in human leukemia K562 cells that served as an adequate model to search for putative involvement of actin cytoskeleton and membrane cholesterol in channel behavior [12,13,22]. In general, our data highlight the putative roles of plasma membrane-cytoskeleton interactions in functional regulations of native Piezo1 in living cells.

MSCa/Piezo channels could mediate cation fluxes implicated in water-salt balance coupled with cell volume regulations under normal and pathophysiological conditions. It is of particular interest to reveal the detailed roles of Piezo1 channels in blood malignancies compared to healthy blood cells or non-transformed counterparts. To date, little is known about the specific impact of Piezo1 activity on physiological responses in cells of blood origin. In erythrocytes, Piezo1 channels were reported to participate in maintaining volume homeostasis [23], and gain-of-function autosomal dominant Piezo1 mutation caused dehydrated hereditary stomatocytosis [24]. As the K562 cell line could be routinely induced to erythroid differentiation in vitro [25], possible implications of Piezo1 in the cell volume regulation of human leukemia cells might be specially addressed in the future.

In our study, using a specific whole-cell patch-clamp assay as an ultra-precise approach to monitoring single currents in the plasma membrane of K562 cells, we were able to record reliable Piezo1 activity in response to Yoda1 concentration lower than 1 µM under physiological conditions (Figure 3). Of note, in calcium imaging experiments on HEK293 cells with overexpression of hPiezo1, almost no fluorescent signal was observed in response to Yoda1 concentrations lower than 3–4 µM [9]. It should be specifically pointed out that fluorescent Ca^2+^ measurements could detect integral changes in [Ca^2+^]_i_ rather than miniature local calcium fluxes. The selective chemical agonist Yoda1 is widely used to probe the potential impact of Piezo1 on various cellular processes. The Yoda1 concentration range (0.5–2.5 µM) detected in the current study could be taken into account in searching for specific Piezo1-dependent cellular responses to minimize the potential side effects of the agonist. Moreover, applied whole-cell single-current analysis uncovers promising approaches to study native ion channels in the plasma membrane with the use of pharmacological tools.

## 4. Materials and Methods

### 4.1. Cell Culture and Reagents

Human myeloid leukemia K562 cells were obtained from the Russian Cell Culture Collection (Institute of Cytology, St. Petersburg, Russia). Cells were cultured in RPMI-1640 medium containing 10% fetal bovine serum (Biolot, St. Petersburg, Russia) and 80 mg/mL antibiotic gentamicin in a humidified incubator (5% CO_2_ at 37 °C). Before the experiments, the cells were plated on glass coverslips coated with poly-DL-lysine (Sigma-Aldrich, St. Louis, MO, USA, Cat. no. P4158). Yoda1 (Tocris, Abingdon, United Kingdom, Cat. no. 5586) and Jedi2 (Tocris, Abingdon, United Kingdom, Cat. No. 6614) were dissolved in DMSO to obtain a stock solution of 10 mM and 60 mM, respectively. GsMTx4 (Tocris, Abingdon, United Kingdom, Cat. No 4912) was dissolved in dH_2_O to obtain a stock solution of 1 mM. Working solutions of Yoda1, Jedi2 and GsMTx4 were prepared immediately before the experiments. 

### 4.2. RT-PCR

The total RNA and first-strand cDNA were obtained as described earlier [26]. As a negative control, a PCR mixture without MMLV reverse transcriptase was used. The primer sequences (designed using the *GeneRunner* *v5.0.59* software, www.generunner.net, accessed date 19 September 2019) for hPIEZO1 were: 3′-CCAGAACAGGTATCGGAAG-5′ (reverse) and 5′-TGCTGTACCAGTACCTGCTG-3′ (forward); the expected amplicon length is 360 bp. The designed primers spanned an intron at the genomic level to avoid false-positive results from potential genomic contamination of the probes. The cycling conditions were as follows: 9 min 30 sec at 94 °C; 38 cycles of 40 s at 94 °C, 30 s at 65 °C and 30 s at 72 °C; 5 min at 72 °C. PCR reaction (total volume of 15 µL) contained: cDNA (1:3 dilution), 0.5 µM of each primer, 1 x Hot-Tag polymerase buffer (Silex, Moscow, Russia), 250 µM dNTPs, 130 µM MgCl_2_ and 1 unit Hot-Tag polymerase. In total, 10 µL of the reaction was subjected to electrophoresis in 1.5% agarose gel, stained with GelRed Nuclear Acid Stain (1:10,000 dilution, Biotium, Fremont, CA, USA, Cat. no. 41002) and visualized by UV fluorescence.

### 4.3. Immunofluorescence

Immunofluorescent staining was performed at room temperature (RT) unless otherwise stated. The cells were fixed in 4% paraformaldehyde (10 min) and permeabilized with 0.25% Tween-20 (15 min). Nonspecific binding of antibodies was blocked by incubation of the cells in 1× PBS containing 10% goat serum (1 h). Then, the cells were incubated with primary anti-Piezo1 antibodies (1:100 dilution, Novus Bio, Abingdon, United Kingdom, Cat. no. NBP1-78537) overnight at 4 °C. Staining with fluorescently labeled secondary antibodies (goat anti-rabbit (GAR) conjugated to Cy3, dilution 1:200, Santa Cruz, Dallas, TX, USA) was performed in the dark (1 h). Nuclei were counterstained with DAPI (10 min). After each step of staining, cells were washed two to three times with 1x PBS. The cells were mounted on microscope slides using a vectashield mounting medium (Vector Laboratories, Burlingame, CA, USA, Cat. no. H-1000). Samples were visualized on an Olympus FV 3000 confocal microscope (Olympus, Shinjuku, Tokyo, Japan) using a 40×/1.25 NA oil objective. The specificity of anti-Piezo1 antibodies was confirmed by pre-incubation of primary antibodies with control blocking peptide (Novus NBP1-78537PEP, 2 h, 1:3 ratio, Figure 1B) or by incubation of the cells only with secondary GAR-Cy3 antibodies (data not shown). In both cases, no fluorescent signal was observed. Images were processed in ImageJ 1.52S software (U. S. National Institutes of Health, Bethesda, MD, USA, https://imagej.nih.gov/ij/, accessed date 15 October 2019).

### 4.4. Electrophysiology

#### 4.4.1. Patch-Clamp Setup

Ion currents were recorded using an Axopatch 200B operational patch-clamp amplifier (Molecular Devices, San Jose, CA, USA) and an Axon Digidata 1550A (Molecular Devices, San Jose, CA, USA) analog-digital converter. Single currents were recorded using cell-attached and whole-cell configurations of the patch-clamp technique. Pipettes were pulled from borosilicate glass capillaries (BF-150-86-10, Sutter Instruments, Novato, CA, USA) to a resistance of 8–12 MOhm or 3–5 MOhm when filled with relevant solutions for cell-attached or whole-cell, respectively. All experiments were performed at RT on the stage of an inverted microscope with Nomarski optics. Data acquisition was performed using Axon PClamp 10.7 Software Suite (Molecular Devices LLC, San Jose, CA, USA) and SciDAVIs 2.3 (http://scidavis.sourceforge.net, accessed date 8 October 2015).

#### 4.4.2. Solutions

All reagents used for the preparation of working solutions were purchased from Sigma-Aldrich, USA. For cell-attached experiments, recording micropipettes were filled with standard Na^+^-contained external solution that contained (in mM): 145 NaCl, 2 CaCl_2_, 1 MgCl_2_ and 10 HEPES/TrisOH. Potassium bath solution containing (mM): 145 KCl, 2 CaCl_2_, 1 MgCl_2_ and 10 HEPES/TrisOH was used to nullify resting membrane potential. For whole-cell experiments, pipettes were filled with a cytosol-like solution containing (mM) 140 KAsp, 5 NaCl, 2 EGTA/KOH,1 MgCl_2_, 20 HEPES/TrisOH with 0.176 CaCl_2_ to establish free ionized calcium concentration [Ca^2+^]_i_ at 10 nM, thus preventing the activity of Ca^2+^-dependent channels in the cell membrane [26]. The chamber was filled with the standard Na^+^-contained external solution (see above). To block Piezo1 currents, we included gadolinium chloride (10 µM), a known non-selective MSCa channel blocker [27], in the standard external solution. To remove any permeable cations, the external solution with non-permeable NMDG^+^ (N-methyl-D-glucamine chloride, 145 mM NMDGCl, 10 HEPES/TrisOH) was utilized. The pH of all solutions was maintained at 7.3. 

#### 4.4.3. Data Analysis and Statistics

The channel recordings were processed and analyzed in Clampfit 10.7 software (part of Axon PClamp Software Suite, Molecular Devices LLC, San Jose, CA, USA). Recordings were filtered in Clampfit 10.7 using a low-pass Gaussian filter, (−3 dB cutoff frequency is 170 Hz) followed by the removal of possible electrical interference (reference frequency is 50 Hz). Single-channel amplitudes were measured from current records manually using ‘cursors’ tools and (where applicable) from amplitude histograms fitted with multi-peak Gaussian distributions. Particularly, the distance between the centers of neighboring peaks indicates the amplitude of single-channel openings. The obtained amplitude values were additionally verified using the ‘Event detection—Single channel search’ option embedded in Clampfit 10.7 software. Single-channel conductance values were defined by the slope of the current-voltage relationship (I-V) after linear I-V approximation. Averaged conductance values are given as the mean ± S.D. (n—number of experiments). The integral channel activity was estimated as NPo, where Po is the probability of the channel to be open, and N is a number of functional channels in the patch [28]. NPo was determined using the following equation: NPo = $\frac{I}{i\;}$, where I is a mean current on the interval, and i is the unitary current amplitude. 

## Data Availability

Not applicable.

## Supplementary Materials

The following are available online at https://www.mdpi.com/article/10.3390/ijms22157839/s1, Figure S1: Single channel activity of Piezo1 induced by selective channel activator Jedi2, Figure S2: Peptide toxin GsMTx4 prevented Piezo1 activation by Yoda1 in plasma membrane of K562 cells.
