# Supplementary material for: Selective Chemical Activation of Piezo1 in Leukemia Cell Membrane: Single Channel Analysis
Source: Int J Mol Sci. 2021 Jul 22;22(15):7839. doi: 10.3390/ijms22157839 (PMC8346046; doi:10.3390/ijms22157839)
Supplement: Supplementary file 1 [file ijms-22-07839-s001.zip › ijms-1287779-supplementary 2.pdf]

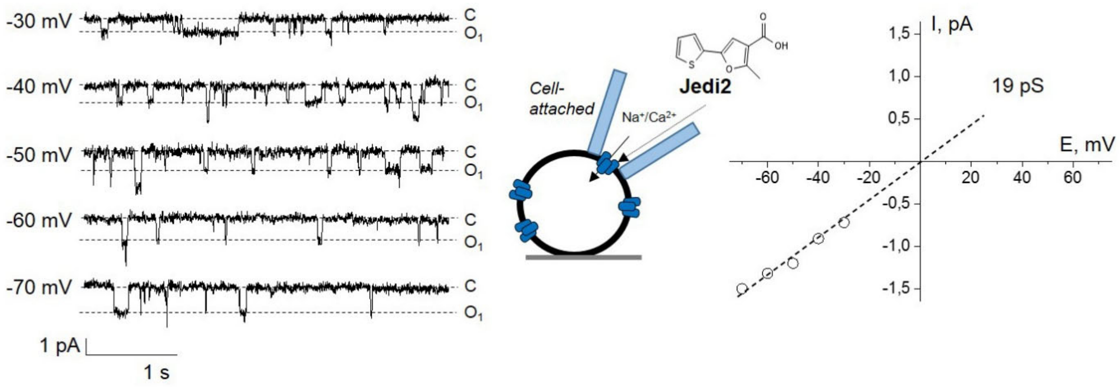

Figure S1. Single channel activity of Piezo1 induced by selective channel activator Jedi2 (6 μM in the pipette solution); current records at different membrane potentials from representative cell-attached experiment (out of 3) on K562 cells. The I-V relationship corresponds to single channel conductance of 19 pS.

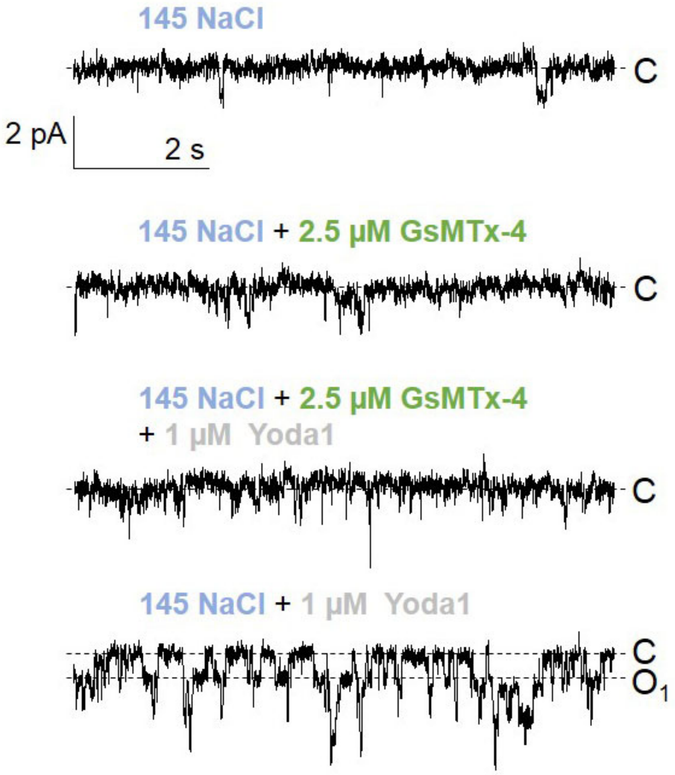

Figure S2. Peptide toxin GsMTx4 prevented Piezo1 activation by Yoda1 in plasma membrane of K562 cells. Representative whole-cell recordings at step-by-step substitutions of extracellular bath solution. No channel activity was observed in the simultaneous presence of GsMTx4 and Yoda1 whereas following washout of the toxin with Yoda1-containing standard extracellular solution induced the activation of Piezo1 channels in the membrane. Holding membrane potential is -40 mV.
